# Study on Tar Generated from Downdraft Gasification of Oil Palm Fronds

**DOI:** 10.1155/2014/497830

**Published:** 2014-01-02

**Authors:** Samson Mekbib Atnaw, Soo Chuan Kueh, Shaharin Anwar Sulaiman

**Affiliations:** ^1^Department of Mechanical Engineering, Universiti Teknologi PETRONAS, 31750 Tronoh, Perak, Malaysia; ^2^Department of Chemical Engineering, Lehigh University, Bethlehem, PA, USA

## Abstract

One of the most challenging issues concerning the gasification of oil palm fronds (OPF) is the presence of tar and particulates formed during the process considering its high volatile matter content. In this study, a tar sampling train custom built based on standard tar sampling protocols was used to quantify the gravimetric concentration of tar (g/Nm^3^) in syngas produced from downdraft gasification of OPF. The amount of char, ash, and solid tar produced from the gasification process was measured in order to account for the mass and carbon conversion efficiency. Elemental analysis of the char and solid tar samples was done using ultimate analysis machine, while the relative concentration of the different compounds in the liquid tar was determined making use of a liquid gas chromatography (GC) unit. Average tar concentration of 4.928 g/Nm^3^ and 1.923 g/Nm^3^ was obtained for raw gas and cleaned gas samples, respectively. Tar concentration in the raw gas sample was found to be higher compared to results for other biomass materials, which could be attributed to the higher volatile matter percentage of OPF. Average cleaning efficiency of 61% which is comparable to that of sand bed filter and venturi scrubber cleaning systems reported in the literature was obtained for the cleaning system proposed in the current study.

## 1. Introduction

**Figure 1:**
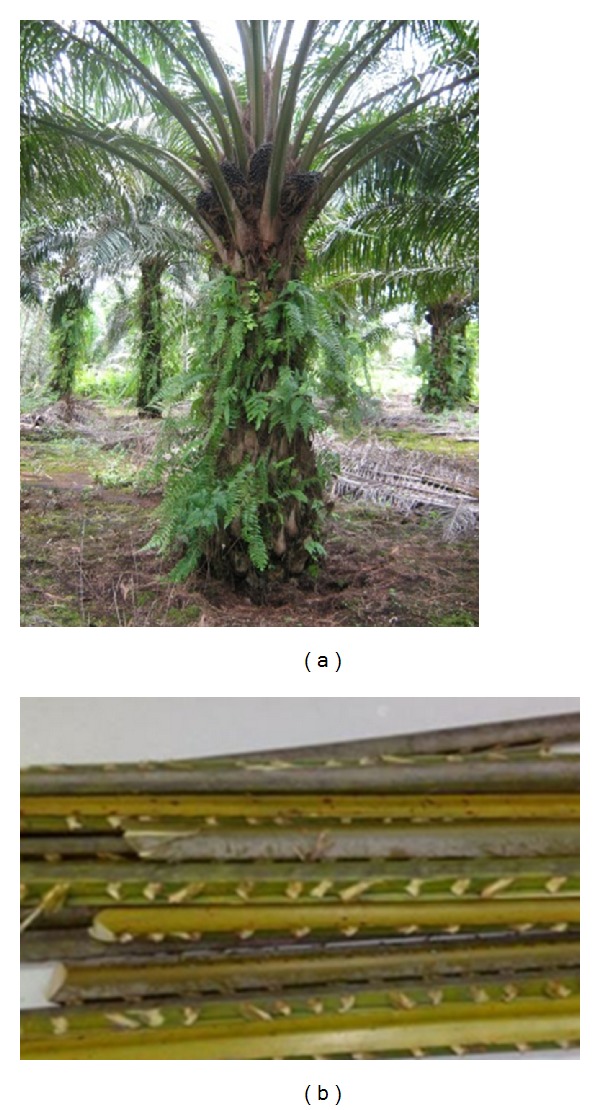
Oil palm biomass (a) picture of oil palm tree, (b) oil palm fronds.

Currently, agricultural and domestic wastes make up a major part of biomass sources. However, the utilization of biomass energy is very small as compared to the conventional ones. Some barriers could be due to issues in policy making, society influence, and readiness of the biomass sources. Malaysia, being one of the biggest global producers and exporters of oil palm, has significantly large plantation areas in the country [1]. Shown in Figures 1(a) and 1(b) are photographs of palm tree and freshly trimmed green OPF, respectively. Most of the parts of the oil palm trees are commercially utilized. However, this is an exception for the fronds, which currently have very limited usage [2, 3]. The fronds are normally left in a huge quantity to naturally decompose on the ground between palm trees for soil conservation, erosion control, and nutrient recycling [4, 5]. By using a proper technology like gasification, there is an opportunity for Malaysia to generate considerable amount of energy from OPF waste.

Studies and experimental data on gasification of oil palm biomass are limited and mostly focused on biodiesel extraction [6–8]. However, oil palm fuels like OPF are expected to generate significant amount of tar, considering their higher volatile matter content (>83%) [4, 9]. Observations from preliminary studies on downdraft, updraft, and high temperature gasification of OPF carried out in the biomass energy research laboratory of University Teknologi PETRONAS indicated generation of significant amount of tar with syngas. There is a huge concern over the level of production of tar in syngas from gasification because tars and particulates are problematic in integrated biomass gasification systems. They may also condense on valves and fittings within the system, thus hampering the ability of valves to function properly. Therefore, it is crucial to develop a systematic approach towards sampling and quantification of tar in biomass producer gases [10, 11]. This current study focuses on measurement of tar concentration in syngas resulting from downdraft gasification of OPF. A tar sampling train custom designed and developed based on a standard tar sampling protocol was used to quantify the gravimetric concentration of tar (g/Nm^3^) in syngas. The amount of char, ash, and solid tar produced from the gasification process was measured in order to account for the mass and carbon conversion efficiency for downdraft gasification of OPF. In addition, the cleaning efficiency of the proposed syngas cleaning system was determined by comparing the tar concentration in raw and cleaned gas samples.

## 2. Methodology

### 2.1. Materials

**Figure 2:**
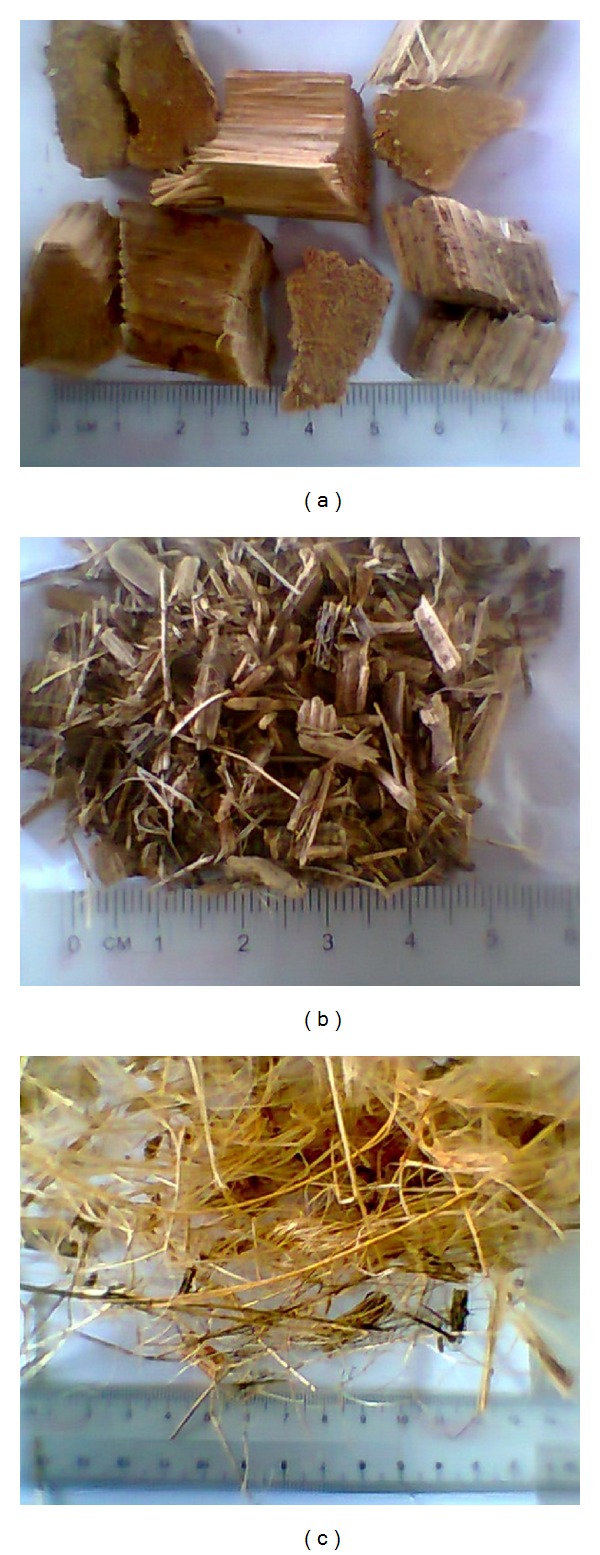
OPF feedstock particle sizes resulting from chipping (a) blocks 20 mm × 20 mm × 20 mm, (b) small particles of size less than 10 mm, (c) fine fibres.

Fresh feedstock of oil palm fronds was obtained from a nearby oil palm plantation several days before the gasification run was carried out. The OPF were collected from the floor of the plantation and brought back to the lab for further processing such as slitting, followed by chipping into the desirable sizes, and lastly drying. Shown in Figure 2 are the different sized particles produced by the chipping machine.  Figure 2(a) shows the block particles that have average dimensions of about 20 mm × 20 mm × 20 mm, which are more suitable for fixed bed gasification. Shown in Figures 2(b) and 2(c) are the small particles of dimension lower than 10 mm and the fibres, respectively. The fibres (Figure 2(c)) with long extremities would cause entanglement of the fuel bed and affect the smooth down flow of the fuel inside the reactor, creating bridging. The particles of smaller dimension (Figure 2(b)) also affect circulation of gasification medium in the reactor and increase pressure drop. Hence, both the fibres and small particles of length lower than 10 mm were not found suitable for gasification. Only the block particles shown in Figure 2(a) were used for gasification. The fuel was processed from green OPF and was predried to achieve moisture content of 18 ± 2%. The ultimate and proximate analysis results of OPF and its heating value are shown in Table 1 [9].

### 2.2. Experimental Setup

**Figure 3:**
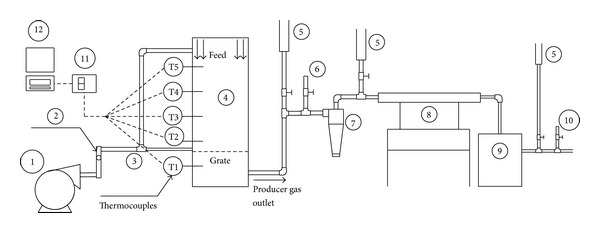
Schematics of the experimental setup: (1) air blower, (2) rotameter, (3) air distribution line, (4) downdraft gasifier, (5) gas flare points, (6) raw gas ampling point, (7) cyclone for gas cleaning, (8) cooling heat exchanger, (9) oil bath filter, (10) clean gas sampling point, (11) temperature data logger, (12) computer.

The gasifier used for the experiment was a laboratory-scale stationary, batch-operated 50 kW fixed-bed downdraft gasifier. The schematic of the experimental setup is shown in Figure 3. Atmospheric air was supplied into the gasifier using a 250 W vortex blower and the amount of air supplied was controlled using a ball valve and a bypass point and monitored using a Pitot tube and a water manometer. The downdraft gasification of OPF was conducted controlling the inlet air flow into the gasifier at an average value of 200 lpm that keep the reactor temperature in between 700 and 900°C. Various syngas conditioning units were provided downstream of the gasifier for the cooling and cleaning of syngas, which include a cyclone (7), cooling heat exchanger (8), and oil bath filter (9) which were provided before the cleaned gas sampling point (10). In addition, as shown in Figure 3, a number of gas flare points (5) were provided on the outlet piping in order to check the combustibility of produced syngas and to burn poisonous gases like CO before being released to the atmosphere.

**Figure 4:**
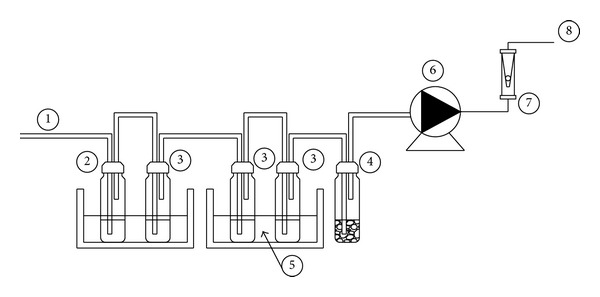
Schematics of gas sampling train: (1) gas from sampling line, (2) moisture collector, (3) series of impinge bottles, (4) backup VOC adsorber (5) ice bath at −20°C, (6) suction pump, (7) rotameter for measuring sampled gas flow rate, (8) gas outlet.

Shown in Figure 4 is schematic of the gas sampling train depicting its various component parts. The gas sampling train consisted of a short and small volume sampling line (1) to avoid clogging due to condensation, a number of impinge bottles (3) filled with proper solvent for tar trapping and a suction pump (6) and flow measuring rotameter (7). The first two impinge bottles filled with 50 mL of isopropylene alcohol (99% concentration) were placed inside the first reservoir containing water at room temperature. In the first impinge tube (2) moisture was condensed by absorption in isopropynol alcohol, in which the heat released by cooling and condensation was removed by the water bath at 20°C surrounding the impinger bottles. After the first moisture collector bottle the gas was passed through three more liquid tar collecting impingers (3), the last two of them immersed in a cooling liquid (ice, water, and salt mixture) at −20°C. A final backup volatile organic component (VOC) sampler impinge bottle (4) filled with activated carbon (used as adsorbent) was used to collect residual solvent that passed the other impingers as shown in Figure 4. The temperature of cooling liquid and sizing and specifications of equipment was determined based on guidelines developed for sampling and analysis of tar in biomass syngas [12, 13]. Suction/peristaltic pump (6) of capacity 0.167 lpm induced the flow of gas through the sampling train. The flow rate of the sampled gas for the duration of sampling was measured using a rotameter (7). The measured flow rate was used to obtain the amount of tar produced per unit volume of sampled gas [g/Nm^3^].

The sampling line was connected to the syngas outlet only after normal operation temperature of the gasifier was achieved and after ensuring productions of a stable flare. Gas sampling was done by connecting the gas sampling train to the syngas outlet pipe until adequate quantity of tar was collected, while recording the sampling duration using a stop watch. After sampling was completed, all the impinger bottles and connecting tubes were rinsed with isopropanol solvent and the sample was collected in a single flask and heated to 83°C for about 45 minutes till all the isopropylene solvent was evaporated. The heating was done making use of a rotary vacuum evaporator to separate tar from the isopropanol tar mixture. Finally, the amount of tar collected was measured using a scale of milligram (mg) accuracy. In addition to the tar samples from the sampling train, tar and condensate collected from the cyclone and outlet pipe lines were also weighed and properly stored for further analysis. The chemical composition of tar samples collected from the sampling train as well as samples of condensate and tar collected from outlet pipe lines and cyclone were analyzed using Agilent 7890A gas chromatography, 5979 C MS. For the case of tar sample from the sampling train, the GC results were normalized after removing the solvent peak, while the tarry condensate collected from the cyclone and syngas outlet pipelines was analyzed directly. The GC methods and conditions used for analysis of the tar are shown in Table 2.

## 3. Results and Discussions

### 3.1. Gasification Results

**Figure 5:**
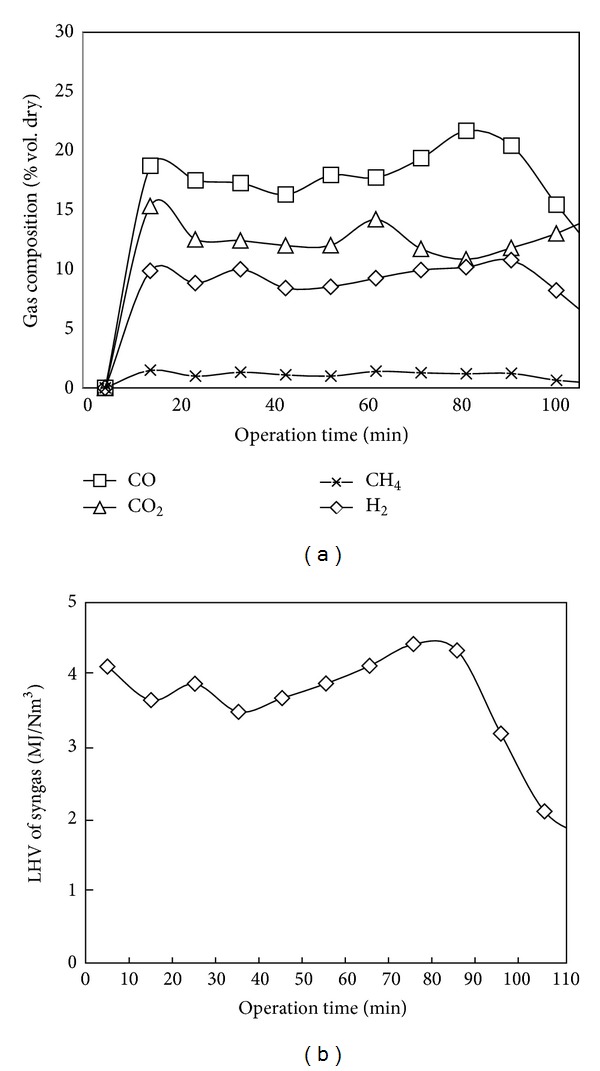
Variation of gasification output with operation time (a) syngas composition, (b) syngas heating value.

Shown in Figure 5(a) is the variation of syngas composition with operation time for gasification experiment with 12 kg of fuel and initial moisture content of 18 ± 2% on wet basis loaded to the gasifier operating at an average inlet air flow rate of 200 lpm. The total operation time was 110 minutes with a fuel feed rate of 6.55 kg/hr. The lower heating value of syngas produced from gasification was estimated based on composition percentage and heating values of the major fuel gas components: CO, H_2_ and CH_4_. The following relation, which was widely used in the literature [14, 15], was used to calculate LHV of syngas:

(1) $$\begin{array}{c} {\left(\text{LHV}\right)}_{\text{CO}}\times X_{\text{CO}}+{\left(\text{LHV}\right)}_{{\text{H}}_{2}}\times X_{{\text{H}}_{2}}+{\left(\text{LHV}\right)}_{\text{C}{\text{H}}_{4}}\times X_{\text{C}{\text{H}}_{4}}, \end{array}$$

where the LHV of the major fuel components of syngas, CO, H_2_, and CH_4_, was taken to be 13.1 MJ/Nm^3^, 11.2 MJ/Nm^3^, and 37.1 MJ/Nm^3^, respectively [14, 15]. The values of *X*_CO_, *X*_H_2__, and *X*_CH_4__, represent the dry volume percentage of each component obtained from measurement of syngas composition. From Figures 5(a) and 5(b) it can be seen that the concentration of the different component gases produced and the heating value of syngas showed an increase in the first few minutes of operation during startup. As can be seen from the figures the steady operation duration was the duration between the 10th minute up to the 80th minute and both concentration of gas components and heating value decreased after the 80th minute because of depletion of the batch of fuel fed to the reactor. The oxidation zone temperature varied in the range between 800 and 1200°C for the duration of optimum operation between the 10th and 80th minutes with an average value of 773°C. The average values of temperature in the different gasification zones, concentration of gas components (CO, CO_2_, CH_4_, H_2_, and N_2_), and heating value of syngas taken over the steady operation duration are shown in Table 3. The temperature values of the different gasification zones, gas composition results, and average heating value of syngas shown in Table 3 are found to be within the range of results reported in the literature for downdraft gasification other biomass fuels.

Direct measurement of the flow rate of syngas produced from gasification was found to be a difficult task due to lack of gas flow rate measuring equipment that could work at the high temperature of syngas exiting the gasifier. Moreover, the exposure of flow measuring units like Pitot static tubes and Rotameter units to the tar in syngas was observed to affect the accuracy of flow measurement due to tar condensation inside the tubes and flow paths of the measuring units. As a result various authors [7, 13, 16] suggested indirect estimation of the syngas flow yield considering either carbon balance or nitrogen balance of the entire gasification system. The syngas yield per unit mass of fuel (Nm^3^/kg) was estimated using the Nitrogen balance method for estimation of specific syngas yield, *Y*_*g*_ using the relation [7, 16, 17]

(2) $$\begin{array}{c} Y_{g}=\frac{Q_{a}\times 0.79}{W_{c}\times {\text{N}}_{2}\%}, \end{array}$$

where *Q*_*a*_ is inlet air flow rate (Nm^3^/hr), *W*_*c*_ is biomass feed rate (kg/hr), and N_2_% is the volume fraction of N_2_ in dry product gas. Hence, the flow rate (Nm^3^/hr) of syngas, *Q*_*g*_ could be calculated as

(3) $$\begin{array}{c} Q_{g}=Y_{g}\times W_{c}, \end{array}$$

where *Y*_*g*_ is specific gas yield per unit mass of fuel and *W*_*c*_ is biomass feed rate (kg/hr). The cold gas efficiency, *η*_th_ of gasification, was given in various literatures as shown in the following relation [18–20]:

(4) $$\begin{array}{c} \eta_{\text{th}}=\frac{{\text{LHV}}_{\text{gas}}\times Q_{g}}{H_{\text{fuel}}\times W_{c}}\times 100, \end{array}$$

where *η*_th_ is cold gas thermal efficiency (%), LHV_gas_ is average lower heating value of syngas (MJ/Nm^3^), *Q*_*g*_ is the syngas generation rate (Nm^3^/hr), *H*_fuel_ is the lower heating value of fuel (MJ/kg), and *W*_*c*_  is the biomass feed rate (kg/hr).

The carbon conversion efficiency is a major parameter that indicates the level of efficiency of thermochemical conversion of carbonaceous fuels and gives an opportunity to compare the results with the work of other researchers. The following relation was used to calculate the carbon conversion efficiency, *η*_*c*_ of the gasification process [18, 20]:

(5) $$\begin{array}{c} \eta_{c}=1-\;\frac{C_{\text{outlet}\;\text{stream}}}{C_{\text{inlet}\;\text{stream}}}, \end{array}$$

where *C*_outlet stream_ is the total rate of carbon in the outlet stream and *C*_feed stream_ is the total rate of carbon in the feed stream. The ultimate analysis of char generated from OPF gasification was carried out in the lab making use of Leco CHNS-932 unit. Shown in Table 4 is the ultimate analysis result for char produced from three different experimental runs. The average of the carbon concentration in gasification char was used in the calculation of carbon conversion efficiency. From investigation of gasification tar properties of four different biomass species, Li and Suzuki [21] suggested that elemental composition of tar from biomass gasification shows less variation with biomass type. In addition, the study indicated elemental that composition of gasification tar does not significantly vary with operation temperature, with the average values of carbon, hydrogen, and oxygen composition given as 54.5%, 6.5%, and 39% on mass basis, respectively. Therefore, these tar composition values were used in carbon balance calculation in this study.

The byproducts produced from the gasification process (ash, char, and tar) were collected at the end of the gasification experiment and the mass of each product was measured in order to account for the mass and carbon balance of the process. The total amounts of ash, char, and tar produced from gasification process, the flow rate of syngas, and the gas yield estimated based on the Nitrogen balance approach ((2) and (3)) were also shown in Table 5. The gas yield of 2.51 Nm^3^/kg obtained for downdraft gasification of OPF was found to be slightly higher compared to those of empty fruit bunches (2.04 Nm^3^/kg) and sawdust (2.0 Nm^3^/kg) reported in the literature [7], implying the significant potential of OPF biomass as a source of fuel gas generation. The fuel consumption rate of 6.37 kg/hr for downdraft gasification of OPF was also found to be within the typical range of 2.72–9.48 kg/hr reported in the literature [16, 18, 22] for gasification of coal, wood, and hazelnut shells biomass materials in gasifies of similar capacity. Cold gas efficiency, mass conversion, and carbon conversion efficiencies that are acceptable for gasification were also obtained. The calculated cold gas efficiency of gasification accounts for the heating value of unconverted carbon and volatile matter in produced tar, char, and ash [17]. The gasification results showed that OPF has a high potential to be used as a gasification fuel resulting in comparable output and performance with other biomass materials.

### 3.2. Tar Concentration in Syngas

The concentration of tar in syngas produced from downdraft gasification of OPF was calculated using the relation

(6) $$\begin{array}{c} C_{t}=\;\frac{W_{t}}{V_{g}}, \end{array}$$

where *C*_*t*_ is concentration of tar in syngas (g/Nm^3^), *W*_*t*_ is weight of tar (g) in sampled gas, and *V*_*g*_ is volume of sampled gas (m^3^). The weight of tar in sampled gas (*W*_*t*_) was measured by weighing the residual tar after evaporating the isopropanol alcohol with high precision digital scale. The total volume of sampled gas, *V*_*g*_, was determined from the flow rate of sampled gas measured using a rotameter mounted at the end of the sampling train, and considering the sampling duration recorded using a stop watch. The measured tar concentration for raw gas samples taken before the cooling and cleaning units (cyclone, condenser, and oil bath filter) and that of cleaned gas for three experimental runs are given in Table 6. In addition the cleaning efficiency of the gas conditioning units downstream of the gasifier was calculated and is shown in Table 6. The cleaning efficiency was calculated as the ratio between the difference in tar concentration (before and after cleaning) and the initial tar concentration. Average concentrations of tar in raw gas and cleaned gas samples of 4.928 g/Nm^3^ and 1.923 g/Nm^3^, respectively, were obtained. The amount of tar in raw syngas obtained was found to be higher compared to expected lower level of tar concentration for downdraft gasification system which is reported to be lower than 1–1.6 g/Nm^3^ [23, 24]. This indicates that tar cracking and cleaning are the major challenges in using OPF as a gasification feedstock. The average cleaning efficiency of 61% resulted from the use of the cyclone, condenser, and oil bath filter system was found to be comparable with that of sand bed filter and venturi scrubber cleaning systems reported in the literature [10]. An average cleaned gas tar concentration of 1.923 g/Nm^3^ was obtained. This indicates the need for further cleaning if the syngas is going to be used in applications involving sensitive equipment like IC engines, which require a level of tar concentration lower than 0.05 g/Nm^3^ [25]. Therefore, in future research, application of cleaning systems with better efficiency needs to be investigated.

### 3.3. Tar Component Analysis

The tar and condensates products generated during gasification commonly consisted of acids, alcohols, aldehydes, ketones, easters, heterocyclic derivatives, and phenolic compounds [26]. The composition of tar samples was studied making use of Agilent 7890A GC, 5979 C MS unit. The composition test was carried out for a sample of tar-isopropanol mixture and pure tar sample taken from the cyclone. Mass spectrometry (MS) result of the area percentage of the various tar components indicating the relative area percentage of tar components for pure tar sample and tar-solvent mixture is given in Table 7. After removing the first peak of isopropanol solvent the components area percentage which indicates the relative abundance of each component for pure tar and tar-solvent mixture was found to be comparable. As can be seen from Table 7, the major components found in tar from downdraft gasification of OPF were Acetic acid and Phenol with area percentage of 62% and 12.8%, respectively.

## 4. Conclusions

Gasification of OPF in a downdraft fixed bed reactor was carried out and the amount of tar concentration in the syngas, the heating value of produced gas, and cold gas and conversion efficiencies of the gasifier were studied. A proposed syngas cleaning system consisting of a cyclone, cooling heat exchanger, and oil bath filter was built and tested and average of cleaning efficiency of 61%, which is comparable with other cleaning systems used in the literature, was obtained. However, relatively higher concentrations of tar in raw gas sample of 4.93 g/Nm^3^ and cleaned gas sample of 1.92 g/Nm^3^ were measured for downdraft gasification of OPF. The relatively higher tar generation for gasification of OPF (as compared to only 1–1.6 g/Nm^3^ reported in the literature for woody biomass) could be attributed to its high volatile matter content (83.5% dry weight basis) and further research in more efficient cleaning systems need to be done in future. In addition, the study carried out on performance of downdraft gasification of OPF in terms of the gas yield, cold gas efficiency, mass, and carbon conversion efficiency showed that OPF has a significant potential for use as a source of generating syngas with comparable output compared to coal and other biomass fuels like wood and hazelnut shells.

## Figures and Tables

**Table 1:** Physical and chemical properties of OPF [9].

Proximate analysis [%]*	
Volatile matter (VM)	83.50
Fixed carbon (FC)	15.20
Ash	1.30

Ultimate analysis* [%]	

C	44.58
H	4.53
N	0.71
S	0.07
O**	48.80
HHV [MJ/kg]	17.28

*Dry weight basis, **by difference.

**Table 2:** Description of GC method used for tar analysis.

GC condition	BPX-S (SGE brand) 30 m *X* 0.25 mm ID *X* 0.25 *μ*m capillary column with column flow 1 mL/min
Oven program	Held at 35°C for 2 minutes followed by heating rate of 20°C/min up to 300°C for 45 minutes
Carrier gas	He
Injector	Injector volume of 1 *μ*L, Mode–split type with split ratio of 50 : 1 with split flow of 50 mL/min. Injector port temperature and pressure of 300°C and 6.77 psi

**Table 3:** Gasification results.

Parameters	Experimental values
Moisture content (% wet basis)	18 ± 2
Inlet air flow rate (l pm)	200
Oxidation zone temp. (°C)	773
Reduction zone temp. (°C)	580
Pyrolysis zone temp. (°C)	422
Drying zone temp. (°C)	150
CO composition (vol.%)	17.54
CO_2_ Composition (vol.%)	12.91
CH_4_ Composition (vol.%)	1.15
H_2_ Composition (vol.%)	9.13
N_2_ composition (Vol.%)	59.28
Peak heating value (MJ/Nm^3^)	4.12
Average heating value (MJ/Nm^3^)	3.75

**Table 4:** Ultimate analysis results for char produced from downdraft gasification of OPF.

Components	C	H	N	S
Average	59.592	2.326	0.916	0.093
Std. Dev.	3.53	0.29	0.28	0.06

**Table 5:** Gasification performance.

Parameter	Measured and calculated values
Ash weight (g)	376.33
Char weight (g)	752.67
Tar weight (g)	73.58
Syngas flow rate (Nm^3^/hr)	16.02
Fuel consumption rate (kg/hr)	6.37
Gas yield (Nm^3^/kg)	2.51
Cold gas efficiency (%)	60.37
Mass conversion efficiency (%)	89.98
Carbon conversion efficiency (%)	91.18

**Table 6:** Tar concentrations measured in raw and cleaned syngas and gas cleaning efficiency.

Parameters	Raw gas tar concentration (g/Nm^3^)	Cleaned gas tar concentration (g/Nm^3^)	Cleaning efficiency (%)
Avg.	4.93	1.92	61.0
Std. dev.	0.55	0.29	3.45

**Table 7:** Major tar components detected by MS and their relative area percentage.

Num.	Chemical product	Area percentage (%)	
		Pure tar sample	Tar-solvent mixture
1	Acetic Acid	62.93	62.88
2	Phenol	9.61	12.8
3	2-propanone, 1-hydroxy	6.02	4.04
4	Furfural	3.64	—
5	Butyrolactone	2.81	2.85
6	Methyl alcohol	1.44	1.04
